# Provincial Medical Association

**Published:** 1837-10

**Authors:** 


### PROVINCIAL MEDICAL ASSOCIATION.

[We recommended the following suggestions to the particular attention of the Council of this Society. They are extracted from an article in the "British Annals" by the editor of that clever Journal.]

The Associations principally promote science by bringing kindred minds together, and exciting a salutary enthusiasm in the pursuit of science that communicates itself to individuals which, in other circumstances, would remain indifferent. With these elements, a judicious division of labour, and a rational organization, the Associations will bear abundant fruit. The Council of the Provincial Medical and Surgical Association (which we shall call the Medical Association,) should be divided into sections. There may be (1) an anatomical and physiological section; (2) a section of pathology and practice of physic; (3) a section of surgery; (4) a section of chemistry, botany, and pharmacy; (5) a section of vital statistics; (6) a section of medical police. Each section should meet before the general meeting; papers might be discussed; experiments repeated; formulæ demonstrated; and the results to be submitted to the general meeting determined. Instead of one retrospective address, a brief, rapid report should be read by a person deputed, at the previous annual meeting, from each section. To prevent loss of time, a certain period may be fixed for the report of each section.

We submit these suggestions to the Council of the Medical Association, from a belief that some such organization would increase its efficiency, and from a firm conviction that no one individual can do critical justice to the publications incessantly appearing in every department of medical science. A man may collect and crowd together all the facts that appear novel; the opinions that are most interesting; the labours of men most distinguished; but can he do more? Is his judgment of great value out of the sphere of his own enquiries? Can he weigh rival claims; do justice, and not do injustice? Can he discover the first glimmering of a great truth from an unknown, unexpected source? Impossible. * *

More Associations should be immediately organized. There can be no doubt of their success when conducted in a proper, liberal spirit; and where they do not encroach on each other's territory. Much must depend on the accidental energy and talent of the medical men of a district; but, looking at the geographical position, we should say Associations should be formed at York, to move to Hull and Lincoln; at Newcastle-upon-Tyne, to take in Berwick and Durham; at Appleby or Carlisle; at Shrewsbury, to extend over North Wales and Cheshire. The metropolis is already occupied. The detached Branches retain all they can desire as independent Societies, and gain strength by union; their coalition into one body is necessary, and inevitable.

---

